# Pig jejunal single-cell RNA landscapes revealing breed-specific immunology differentiation at various domestication stages

**DOI:** 10.3389/fimmu.2025.1530214

**Published:** 2025-02-28

**Authors:** Wenyu Fu, Qinqin Xie, Pengfei Yu, Shuang Liu, Lingyao Xu, Xiaowei Ye, Wei Zhao, Qishan Wang, Yuchun Pan, Zhe Zhang, Zhen Wang

**Affiliations:** ^1^ College of Animal Sciences, Zhejiang University, Hangzhou, China; ^2^ SciGene Biotechnology Co., Ltd, Hefei, China; ^3^ Hainan Institute of Zhejiang University, Building 11, Yongyou Industrial Park, Yazhou Bay Science and Technology City, Yazhou District, Sanya, China; ^4^ Key Laboratory of Livestock and Poultry Resources Evaluation and Utilization, Ministry of Agriculture and Rural Affairs, Hangzhou, China; ^5^ Hainan Yazhou Bay Seed Lab, Yongyou Industrial Park, Yazhou Bay Sci-Tech City, Sanya, China

**Keywords:** single-cell RNA sequencing, jejunum, immune cells, domestication, plasma cells

## Abstract

**Background:**

Domestication of wild boars into local and intensive pig breeds has driven adaptive genomic changes, resulting in significant phenotypic differences in intestinal immune function. The intestine relies on diverse immune cells, but their evolutionary changes during domestication remain poorly understood at single-cell resolution.

**Methods:**

We performed single-cell RNA sequencing (scRNA-seq) and marker gene analysis on jejunal tissues from wild boars, a Chinese local breed (Jinhua), and an intensive breed (Duroc). Then, we developed an immune cell evaluation system that includes immune scoring, gene identification, and cell communication analysis. Additionally, we mapped domestication-related clustering relationships, highlighting changes in gene expression and immune function.

**Results:**

We generated a single-cell atlas of jejunal tissues, analyzing 26,246 cells and identifying 11 distinct cell lineages, including epithelial and plasma cells, and discovered shared and unique patterns in intestinal nutrition and immunity across breeds. Immune cell evaluation analysis confirmed the conservation and heterogeneity of immune cells, manifested by highly conserved functions of immune cell subgroups, but wild boars possess stronger immune capabilities than domesticated breeds. We also discovered four patterns of domestication-related breed-specific genes related to metabolism, immune surveillance, and cytotoxic functions. Lastly, we identified a unique population of plasma cells with distinctive antibody production in Jinhua pig population.

**Conclusions:**

Our findings provide valuable single-cell insights into the cellular heterogeneity and immune function evolution in the jejunum during pig at various domestication stages. The single-cell atlas also serves as a resource for comparative studies and supports breeding programs aimed at enhancing immune traits in pigs.

## Graphical Abstract

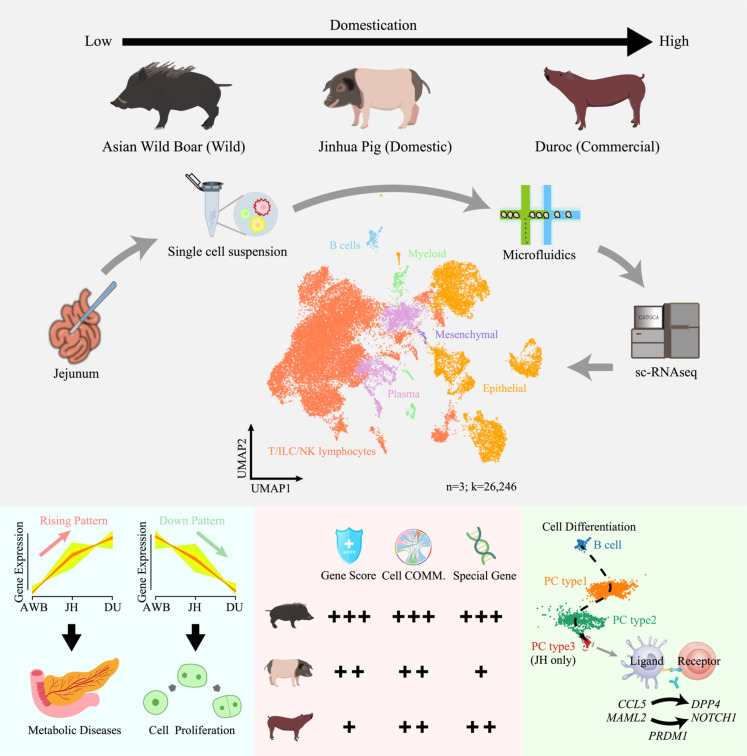

## 1 Introduction

Pigs serve as crucial livestock animals for food production and ideal animal models for biomedical research (1–5). Dietary factors and genetic selection significantly impact pig gut health and immune function (6–8). Domestication has led to genetic and phenotypic diversity, making pigs excellent models for studying genotype-phenotype relationships (9, 10). Geographic differences and human-driven selection have resulted in substantial phenotypic diversity among Eastern (Asian) and Western (European and American) pigs, and wild boars (2, 4). For example, wild boars have shorter digestive tracts (11) and different gut microbiomes, primarily hosting Firmicutes and Actinobacteria, which can produce antibiotics and immune-regulating compounds that enhance disease resistance and nutrient absorption (12). In contrast, domestic pigs’ microbiomes are dominated by Firmicutes and Bacteroidetes, aiding in carbohydrate digestion and energy extraction (12). Moreover, their diversity includes gene abundances linked to metabolism, immune function, and antibiotic resistance. For example, domestic pigs exhibit higher abundances of genes related to carbohydrate metabolism, starch degradation, and a higher diversity of antibiotic resistance genes (ARGs) (13), while wild boars show elevated levels of genes linked to environmental adaptation, immune function, and fiber breakdown. Therefore, by investigating the genetic and phenotypic differences among pigs at different domestication stages, we can gain deeper insights into the key genetic mechanisms, organ function formation, and the improvement of immune traits.

The intestine plays a crucial role in the physiological functions in organism, serving not only as a site for digestion but also as an important component of the immune system (14–17). Different cell types within the intestine play distinct roles, and understanding the characteristics and functions of these cell types can provide important insights into intestinal functions. Immune cells from both the innate and adaptive immune systems play a crucial role in intestinal immunity (18). Plasma cells are essential for adaptive immunity, primarily producing IgA in the intestinal lamina propria to neutralize pathogens (19). They also show adaptability, adjusting to different microbial environments to maintain effective immune responses (20). The jejunal immune system serves as a critical defense line against the invasion of pathogenic microorganisms, playing a key role in health and immune response (21). Research focused on jejunal immune function can provide important clues for understanding the differences in immune responses and jejunal health. Additionally, cellular heterogeneity within jejunal tissues is an area of significant interest (22, 23).

Single-cell RNA sequencing (scRNA-seq), as a high-resolution technique, enables the systematic elucidation of cellular composition, cell lineage trajectories, and gene features of individual cells. For example, scRNA-seq studies have uncovered distinct epithelial subtypes, identified key regulatory factors involved in intestinal stem cell maintenance, and revealed the dynamic interaction between immune and epithelial cells in maintaining gut homeostasis. Emerging studies have also revealed cellular heterogeneity in pig jejunal tissues and immune niches (24, 25). Nowdays, few studies have compared domestication-related breed specificity among pig breeds at the single-cell RNA sequencing level (26). Moreover, there have been no detailed investigations describing transcriptional differences at the single-cell level in the jejunal tissues of pigs at various stages of domestication.

In this study, we aimed to first construct a single-cell atlas of jejunal tissues from pigs across three domestication statuses, then systematically uncover the characteristics of the immune cells, focusing on cell composition and biological functional heterogeneity, particularly within plasma cells. Our findings offer novel insights into the architecture of the immune system and the domestication history of the pig jejunum. Ultimately, our findings provide a foundation for future efforts to enhance pig breeding programs aimed at improving immune traits.

## 2 Materials and methods

### 2.1 Pig intestinal tissue preparation and single-cell RNA sequencing

**Figure 1:**
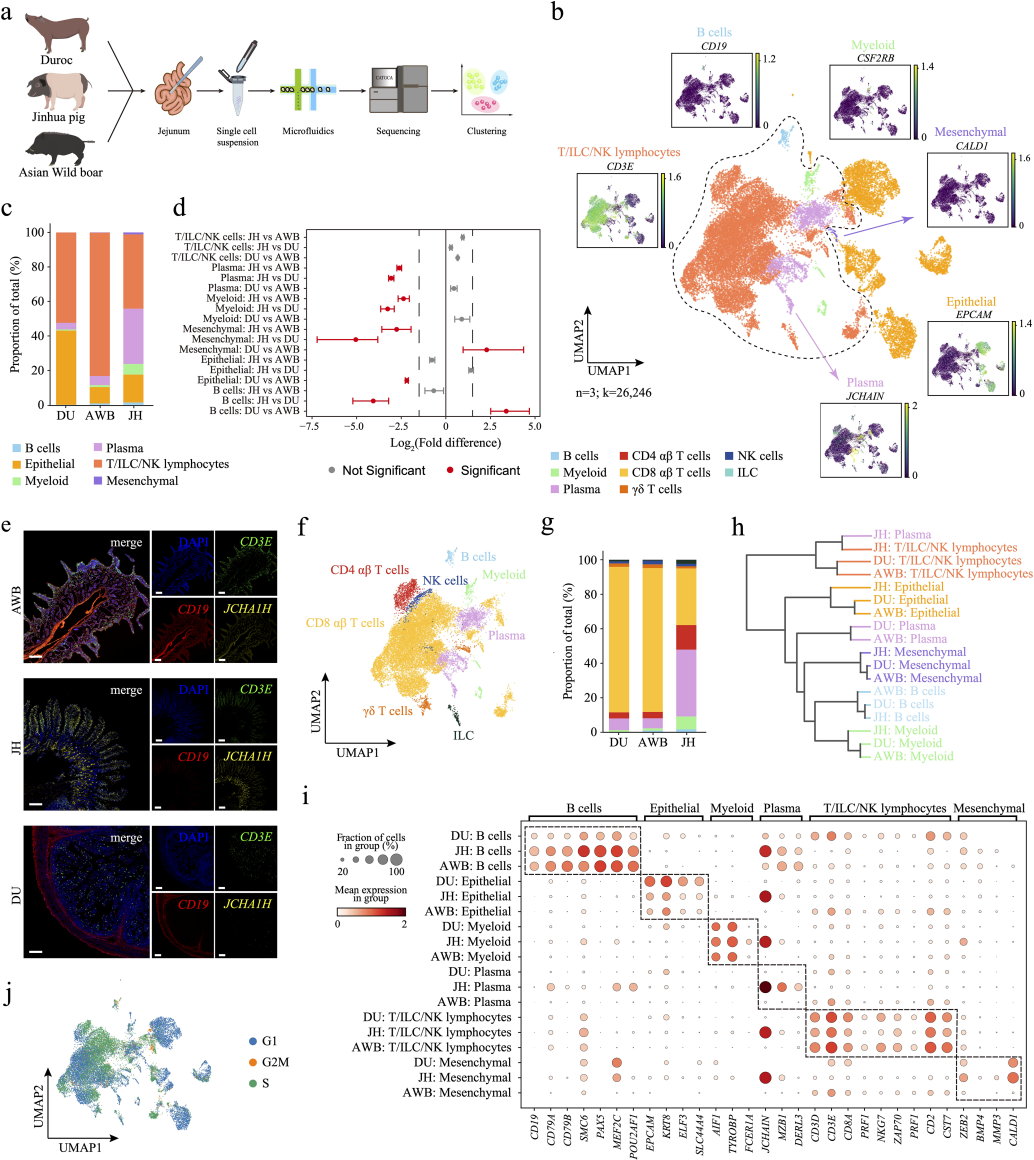
Single-cell transcriptomic analysis reveals the cellular composition and cell cycle dynamics of three porcine jejunal tissues. **(a)** Schematic representation of the sample collection, scRNA-seq, and cross-species analysis workflow. **(b)** UMAP plot showing the expression levels of typical marker genes for various cell populations, with expression intensity indicated in green. Identified populations include epithelial cells, plasma cells, B cells, T/ILC/NK lymphocytes, myeloid cells, and mesenchymal cells. **(c)** Cellular composition plots of the three porcine jejunal cell clusters. **(d)** Relative differences in cell proportions for each cluster between different breeds. Red clusters have an FDR < 0.05 and mean |log_2_(Fold difference)| > 1.5 under comparison (permutation test; n = 1,000). **(e)** Immunofluorescence visualization of the jejunum using markers for *CD3E*+ T/ILC/NK lymphocytes, *CD19*+ B cells, and *JCHAIN*+ plasma cells. Scale bar, 200 μm. **(f)** Immunocytes were further subclustered into ILCs, NK cells, CD4 αβ T cells, CD8 αβ T cells, γδ T cells, plasma cells, B cells, and myeloid cells. **(g)** Composition plot of immune cell types. **(h)** Dendrogram illustrating the similarity of cell types among the three pig species. **(i)** Differential expression patterns of marker genes across different cell types in the three pig species. **(j)** UMAP plot of cells in different cell cycle stages.

Jejunal tissue samples were collected from three healthy adult pigs (Wild boar, Jinhua, and Duroc, **Figure 1a**), representing three domestication statuses: wild boars, local domesticated breed, and an intensive breed for single-cell RNA sequencing. Sample preparation involved placing into gentleMACS C tubes (130-093-237; Miltenyi) containing enzyme digestion solution (Hepes, Liberase TM, and DNase I in HBSS), dissociation using gentleMACS Octo Dissociator (130-095-235; Miltenyi) at 37°C, followed by filtration through a 40-μm filter (352340; BD Falcon), and cell collection via centrifugation (500g for 5 minutes at 4°C). After removing erythrocytes and performing cell counting, the fresh cells were washed twice with Flow Buffer [PBS containing 5% (v/v) FBS and 2 mM EDTA] and then resuspended at a concentration of 1 × 10^6^ cells/mL in 1 × 1640 medium supplemented with 0.04% bovine serum albumin on ice. Following the manufacturer’s protocol, scRNA-Seq libraries were prepared using the 10× Genomics Chromium Single-Cell 3’ kit (V3) and subsequently sequenced using an Illumina NovaSeq 6000 platform at Novogene.

### 2.2 Quality control

Clean reads were obtained from the raw scRNA-seq reads using fastp (27) (v 0.23.1) with default parameters. These clean reads were then aligned to the pig reference Sus scrofa 11.1 (28) to generate a single-cell transcript expression matrix using CellRanger (v 7.0.0) (29) pipeline from 10× Genomics. Subsequently, Scanpy (v 1.9.8) (30) was utilized to process scRNA-seq data from multiple samples. Cell filtering was performed as follows: removed doublet-like cells with doublet scores below 0.25, removed mitochondrial cells with mitochondrial read fraction greater than 50%, and removed cells with fewer than 200 or more than 7500. After these filtering steps, a total of 26,246 cells were included in the downstream analysis.

### 2.3 Power analysis and downsampling test

We performed a power analysis using the scPower website (https://scpower.helmholtz-muenchen.de) (31), setting the number of samples to 3, the cell type frequency to 0.01, the detection power to 0.80, and the minimal number of cells to range from 1 to 21.

We performed a cell number downsampling analysis using the “scanpy.pp.subsample” function in Scanpy (v 1.9.8) (30), reducing the cell count to 2000 for each of the three pig types. Additionally, we performed a sequencing depth downsampling analysis using the “scanpy.pp.downsample_counts” function in Scanpy (v 1.9.8) (30). For this analysis, we selected the average (n=8172) and median (n=6300) cell counts from the DU and AWB samples as “counts_per_cell” values. All downsampling analyses were repeated 5 times. For the obtained downsampled data, we conducted a correlation analysis (Pearson method) between the downsampled data and the original data in pseudobulk level, using the “dc.get_pseudobulk” function in Decoupler (v 1.6.0) (32).

### 2.4 Cell type annotation and proportion test

The final single-cell gene expression matrix was generated by normalizing and scaling the gene counts matrix using Scanpy (v 1.9.8) (30). For cell clustering and type annotation, batch corrections were first performed on 3 libraries using the “sc.external.pp.harmony_integrate” function. Subsequently, genes with top 2000 high variability were identified for dimensionality reduction and Leiden clustering (resolution 0.5-2) using the “sc.tl.leiden” function, resulting in 28 clusters. We selected specific marker genes to distinguish different cell types (**Supplementary Table S1**). Six cell lineages were annotated as followed: epithelial cells [*EPCAM* (33) and *KRT8* (34)], plasma cells [*JCHAIN* (35, 36) and *MZB1* (37, 38)], B cells [*CD19*, *CD79A*, *CD79B*, and *BACH2* (39–41)], T/ILC/NK lymphocytes [*CD3E*, *ZAP70*, *CD4*, *IL7R*, *CD8A*, *GNLY*, *NKG7*, and *PRF1* (40, 42, 43)], myeloid lineage [*ENSSSCG00000028461*, *CD68*, *CSF2RB*, *C1QC*, *APOE*, *FCER1A*, and *KIT* (44, 45)], and mesenchymal cells [*CALD1*, *VIM*, and *ZEB2* (46)]. Additionally, the T/ILC/NK lymphocytes were subdivided into three subtypes (**Supplementary Table S1**): ILCs [*KLRB1*, *CSF2* (47)], NK cells [*PRF1*, *NKG7*, *EOMES* (40)], CD4 αβ T cells [*CD4* (48, 49)], CD8 αβ T cells [*CD8B* (48, 49)], and γδ T cells [*BLK* (48, 49)]. The proportions of each cell type present in each experimental group were tested by bootstrapped permutation tests (1,000 iterations) using scProportionTest package. The comparisons with FDR < 0.05 and mean |log_2_(Fold difference)| > 1.5 were significant.

### 2.5 Cell cycle

Cell cycle scores were calculated using the “sc.tl.score_genes_cell_cycle” function and regressed out the “sc.pp.regress_out” function in Scanpy (v 1.9.8) (30). These scores were based on the geometric mean expression of gene sets representing the G2/M and S phases (Source Data). Cells lacking markers for these phases were categorized as being in the G1 phase. Next, a specialized dataset containing only cell cycle-related genes was generated. Principal component analysis (PCA) and the nearest neighbor graph were then constructed on this refined dataset. After performing regression and normalization, the dataset was visualized using UMAP dimensionality reduction to elucidate cell cycle-associated patterns.

### 2.6 Cell-cell communication

Cell-cell communication analysis was conducted using liana package (v 1.1.0) (50) in Python. The “cellphonedb()” function from the liana.method module was utilized to identify and quantify interactions of ligand-receptor pairs by assessing gene expression correlations (51). The top 20 cell-cell interactions with a significance *P-value* less than 0.01 were visualized through “dotplot()” function.

### 2.7 Immune score estimation

We utilized the “AddModuleScore” function from the Seurat R package (v 4.3.0) (52) to calculate the immune-related or inflammation-related immune scores of each immune cell, covering steps such as mean calculation, matrix partitioning, and background value selection. The immune-related genes were identified through differential expression analysis, while the inflammation-related genes were compiled from the literature (53). The inflammation-related genes included: *GZMB*, *GZMA*, *PRF*, *IFNG*, *IFNGR1*, *ISG20*, *IL4*, *IL4R*, *IL5*, *IL6*, *IL10*, *IRF2*, *IL12B*, *IL17A*, *IL17F*, *IL17RA*, *IL2*, *IL2RB*, *IL21*, *IL21R*, *NFKBIA*, *RORA*, *RORC*, *S100A8*, *S100A9*, *STAT1*, *STAT3*, *STAT4*, *TGFB1I1*, *TNFRSF1B*, and *TNF*.

### 2.8 Measurement of T cell status

We measured the expression levels of specific markers to determine the memory phenotype (*KLRB1* and *IL7R*), tissue-resident (*RUNX3*, *CD69*, *NR4A1*, *CXCR6*, and *CD103*), cytotoxic phenotype (*GZMA*, *GZMB*, *PRF1*, *GNLY*, *CST7*, and *TNFSF10*), exhaustion phenotype (*PDCD1*, *CTLA4*, *HAVCR2*, and *LAG3*), and co-stimulatory (*CD28*, *CD226*, *ICOS*, and *TNFRSF9*) of T cells (53).

### 2.9 Differential expressed gene (DEGs) identification

We used the Wilcoxon rank-sum test method to identify differentially expressed genes with the “scanpy.tl.rank_genes_groups” function in Scanpy (v 1.9.8) (30). DEGs between cell types were defined by adjusted *P-values* (Benjamini-Hochberg method) lower than 0.05 and log_2_FC greater than 1.5.

### 2.10 Clustering categorization and identification of gene under fixed breed sequence

We performed clustering analysis on the gene expression data using the Mfuzz R package (v 2.54.0) (54). The average expression matrix, after standardization, was used for soft clustering with a setting number 9 through the “mfuzz” function. The number of clusters was determined by “Dmin” function. Subsequently, the “mestimate” function was employed to determine the optimal fuzziness coefficient (m-value). We identified DEGs (refer to 2.8) between JH and AWB, as well as between JH and DU. Then we took the intersection of DEGs and Clustering genes to uncover four distinct expression patterns: domestication-increasing expression, JH-high expression, JH-low expression, and decreasing expression.

### 2.11 Functional enrichment analysis

GSEApy package (v 1.1.1) (55) was used to perform functional enrichment analysis. We selected the Gene Ontology (GO) (56) Biological Process database and the Kyoto Encyclopedia of Genes and Genomes (KEGG) (57) genomic resource library as our gene sets. A hypergeometric distributions test was applied to determine whether a gene list enriched in a term or a pathway. Significantly enriched GO terms and KEGG pathways were determined using adjusted *P-values* (FDR method) less than 0.05. To simplify the enrichment analysis results, we utilized the simplifyEnrichment package (v 1.12.0) (58). The “GO_similarity()” function was employed for clustering the enrichment results, and the “simplifyGO()” function was used for visualization.

### 2.12 Pseudotime analysis

We performed pseudotime analysis using Scanpy (v 1.9.8) (30) and employed the Diffusion Pseudotime (DPT) algorithm for B cells and plasma cells. First, we mapped the data to a low-dimensional space using “sc.tl.diffmap” function. Second, we selected B cells as the root cells and computed the transition probabilities between cells iteratively, starting from the root cells. The distance of each cell from the root cells was represented as pseudotime. Third, we visualized developmental trajectory in cell types using “sc.pl.scatter” function.

### 2.13 RNA velocity analysis

We performed RNA velocity analysis using the scVelo package (v 0.2.4) (59). First, we preprocessed the scanpy object data by ensuring trimmed cell indices and adding sample labels steps and integrated the preprocessed data with a loom file. Second, we used the sc.pp.moments” function in Scanpy (v 1.9.8) (30) to compute the moments for the cells. Third, we inferred the dynamic relationships between cells by computing the first-moment matrix (mean) and second-moment matrix (variance). Fourth, we used the “scvelo.tl.velocity” function to compute the velocities of cells. Fifth, we used the “scvelo.tl.velocity_graph” function to calculate cosine correlations. To visualize the integrated data, we generated stream plots using the “scvelo.pl.velocity_embedding_stream” function and colored the UMAP plots with the “scvelo.pl.scatter” function.

### 2.14 Gene regulatory network analysis

We performed gene regulatory network (GRN) analysis using PySCENIC (v 0.12.1) (60). First, we executed the GRN step to establish associations between transcription factors (TFs) and target genes, outputting a CSV file to summarize these relationships. Following this, we further refined our GRN by executing the ctx step to associate motifs with TFs. We generated a heatmap using “sns.clustermap” to illustrate the average area under the curve (AUC) scores for each cell type.

### 2.15 Immunofluorescence

The three jejunal tissues from separate cohorts of pigs (AWB, JH, and DU) were submerged in 4% PFA overnight at 4°C, embedded in paraffin and processed into 5 μm thick sections. After deparaffinization and antigen retrieval (G1202; Servicebio), samples were blocked with 3% BSA for 30 min at room temperature. Afterwards, sections were incubated with the primary antibodies overnight at 4°C. Then, the samples were washed with PBS and incubated with secondary antibodies in the dark for 1 h at room temperature. Next, the washed slides were stained with DAPI and coverslipped with mounting solution (G1221; Servicebio). The following primary antibodies were used: anti-EPCAM (1:200; GB11274; Servicebio), anti-JCHAIN (1:3000; GB111452; Servicebio), anti-CD3E (1:2000; GB12014; Servicebio), and anti-CD19 (1:2000; GB11061-1; Servicebio).

## 3 Results

### 3.1 Single-cell atlas of swine jejunum

We performed droplet-based scRNA-seq to profile the jejunum (JE) of Asian wild boar (AWB, n=1), Jinhua pig (JH, n=1) and Duroc pig (DU, n=1) (**Figure 1a**) and obtained single-cell atlas data from 26,246 cells (AWB=11,929, JH=2,645, and DU=11,672, **Supplementary Figure S1a**). Using Leiden clustering and marker-gene analysis, we annotated six major cell clusters: epithelial cells, plasma cells, B cells, T/ILC/NK lymphocytes, myeloid-like cells, and mesenchymal cells (**Figure 1b**, **Supplementary Figure S1b, d**, **Supplementary Table S1**). The atlas comprised two major cell types—T/ILC/NK lymphocytes (65.4%) and epithelial cells (25.1%)—and four minor cell types—plasma cells (7.3%), myeloid cells (1.4%), B cells (0.6%), and mesenchymal cells (0.2%). We observed heterogeneity in cell composition across pig breeds, particularly among immune cells (**Figure 1c**). The AWB breed enriched for T/ILC/NK lymphocytes (83.0%, **Supplementary Table S2**) than JH and DUR. Specifically, the JH breed was exhibited a higher proportion of plasma cells (32.0%), while the DU breed showed a higher proportion of B cells (43.0%, **Figure 1d**) than other breeds. Additionally, we performed immunofluorescence visualization of the jejunum using markers for *CD3E*+ T/ILC/NK lymphocytes, *CD19*+ B cells, and *JCHAIN*+ plasma cells to experimentally validate the identified cell types and their spatial localization within the jejunum. The results confirmed that these cell types are specifically located in the jejunum (**Figure 1e**). Furthermore, we annotated the T/ILC/NK lymphocytes into five subtypes: ILCs, NK cells, CD4 αβ T cells, CD8 αβ T cells, and γδ T cells (**Figure 1f**, **Supplementary Figure S1e**, **Supplementary Table S1**). Among these subtype cells of T/ILC/NK lymphocytes, the AWB and DU breeds exhibited similar compositional patterns, which were notably distinct from those observed in the JH breed (**Figure 1g**). Obviously, JH displayed a markedly higher proportion of CD4 αβ T cells (14.2%) and lower proportion of CD8 αβ T cells (32.8%) compared to AWB (3.6% and 83.4%, respectively) and DU (3.5% and 84.3%, respectively, **Supplementary Figure S1f**, **Supplementary Table S2**). These cell proportions, especially plasma cells and T cells, highlighting a unique immune cell architecture in JH.

To assess the reliability of our annotated cell types, we conducted GO and KEGG enrichment analyses on differentially expressed genes (DEGs) of each major cell type. The enrichment results confirmed that the identified cell types align well with their respective cellular functions (**Supplementary Table S3**). For instance, DEGs from epithelial cell that plays important roles in absorption were enriched in processes related to nutrient absorption and metabolism, such as fructose catabolic processes and fat digestion and absorption. Plasma cells associated with antibody production crucial for neutralizing pathogens were enriched in pathways like the intestinal immune network for IgA production. B cells involved in immune activities showed enrichment in pathways including primary immunodeficiency and B cell receptor signaling. T/ILC/NK lymphocytes enriched in T-cell-related functions, including the T cell receptor signaling pathway and positive thymic T cell selection. Mesenchymal cells participated in immune regulation and were enriched in pathways such as the regulation of neutrophil degranulation. Furthermore, within the subtypes of T/ILC/NK lymphocytes, CD4 αβ T cell was enriched in pathways like alpha-beta T cell differentiation, highlighting their crucial role in immune responses. ILCs and NK cells play significant roles in intestinal immunity, as evidenced by their enrichment in immune-related pathways such as inflammatory bowel disease and viral protein interactions with cytokines and cytokine receptors. These enrichment analyses substantiate the functional identities of the identified cell types, reinforcing the validity of our single-cell atlas.

Although our dataset varied in cell count and sequencing depth with variety, this dataset was authoritative in explaining cell types (**Supplementary Figure S1g**), as even samples with the smallest cell count were sufficient to explain the current cells (**Supplementary Figure S1h**). To assess the impact of differences in cell numbers and sequencing depth on expression patterns, we performed separate downsampling analysis to subsample both the number of cells and the counts from the count matrix. As expected, we observed the high correlation (>0.9) in gene expression between downsampled counts matrix and the original counts matrix across all three pig groups in each of the three downsampling scenarios (**Supplementary Figure S1i**), supporting the high similarity between downsampled data and our current dataset particularly in terms of gene expression.

### 3.2 Breeds-specific functions of cell type in the jejunum

To assess the transcriptional conservation of cell types across different pig breeds, we identified cell type-specific marker genes in AWB, JH, and DU using single-cell RNA sequencing (scRNA-seq) (**Figure 1i**). By quantifying the preservation of cell-type-specific gene expression programs in jejunal cells, we observed that the jejunum maintains similar overall functionality across the three breeds. For example, T/ILC/NK lymphocytes consistently expressed key marker genes such as *CD3D*, *CD3E*, and *CD2* (**Figure 1i**). This conservation suggests that these core functions are essential for maintaining gut immunity, reflecting a common evolutionary strategy to protect the gut from pathogens and ensure homeostasis. Despite the overall similarity, we detected notable differences in the expression levels of certain cell type-specific genes among the breeds. In epithelial cells, AWB and JH exhibited similar expression patterns, whereas DU showed elevated expression of epithelial cell markers like *EPCAM* and *KRT8* (**Figure 1i**, **Supplementary Figure S1d**). This increase in DU may be attributed to long-term artificial selection and genetic improvement (61). Additionally, AWB and JH had higher expression levels of B cell markers such as *PAX5* and *MEF2C* compared to DU. Notably, JH displayed significantly higher expression of plasma cell markers, including *JCHAIN*, *MZB1*, and *DERL3*, which correlates with their increased proportions of both B cells and plasma cells. This suggests that JH may rely more heavily on humoral immunity mediated by these cells (62).

To further elucidate the similarities and differences among cell types, we performed hierarchical clustering to generate a dendrogram (**Figure 1h**). The dendrogram indicated that most cell types from the three breeds clustered together, highlighting a general similarity in their cellular profiles. However, plasma cells from JH distinctly separated from those of AWB and DU and were more closely related to JH’s T/ILC/NK lymphocytes. This separation suggests that JH plasma cells possess a unique gene expression profile.

Next, we evaluated the impact of cell cycle phases on our clustering results and analyzed cell proliferation by conducting cell cycle analysis and generating a UMAP plot (**Figure 1j**). The distribution of cells across different cell cycle phases was even, indicating that cell cycle genes did not significantly influence the clustering outcomes. Additionally, cell cycle distribution maps for each breed revealed similar patterns in T/ILC/NK lymphocytes but distinct heterogeneity in other cell types, including B cells, myeloid cells, plasma cells, and epithelial cells (**Supplementary Figure S2a**). Specifically, B cells in AWB were predominantly in the G2M and S phase rather than the G1 phase (**Supplementary Figure S2a**), suggesting active cell division, possibly in response to external stimuli. This finding aligns with the previously observed high expression of B cell marker genes in AWB pigs (**Supplementary Figure S2a**). In contrast, myeloid cells and plasma cells in JH were mostly in the G1 phase rather than the S phase (**Supplementary Figure S2a**), indicating that these cells are primarily in a growth and functional state rather than actively replicating DNA and preparing for division. This observation implies that the heterogeneity in JH plasma cells is not due to abnormal proliferation caused by antigenic stimulation. Additionally, epithelial cells in DU predominantly resided in the G1 phase, suggesting that these cells are mainly engaged in growth and functional activities rather than cell division.

Overall, these analyses demonstrate both the conserved and breed-specific transcriptional profiles of jejunal cell types, providing insights into the immune architecture and potential breed-specific adaptations in pigs.

### 3.3 The conservation and heterogeneity of immune cells

**Figure 2:**
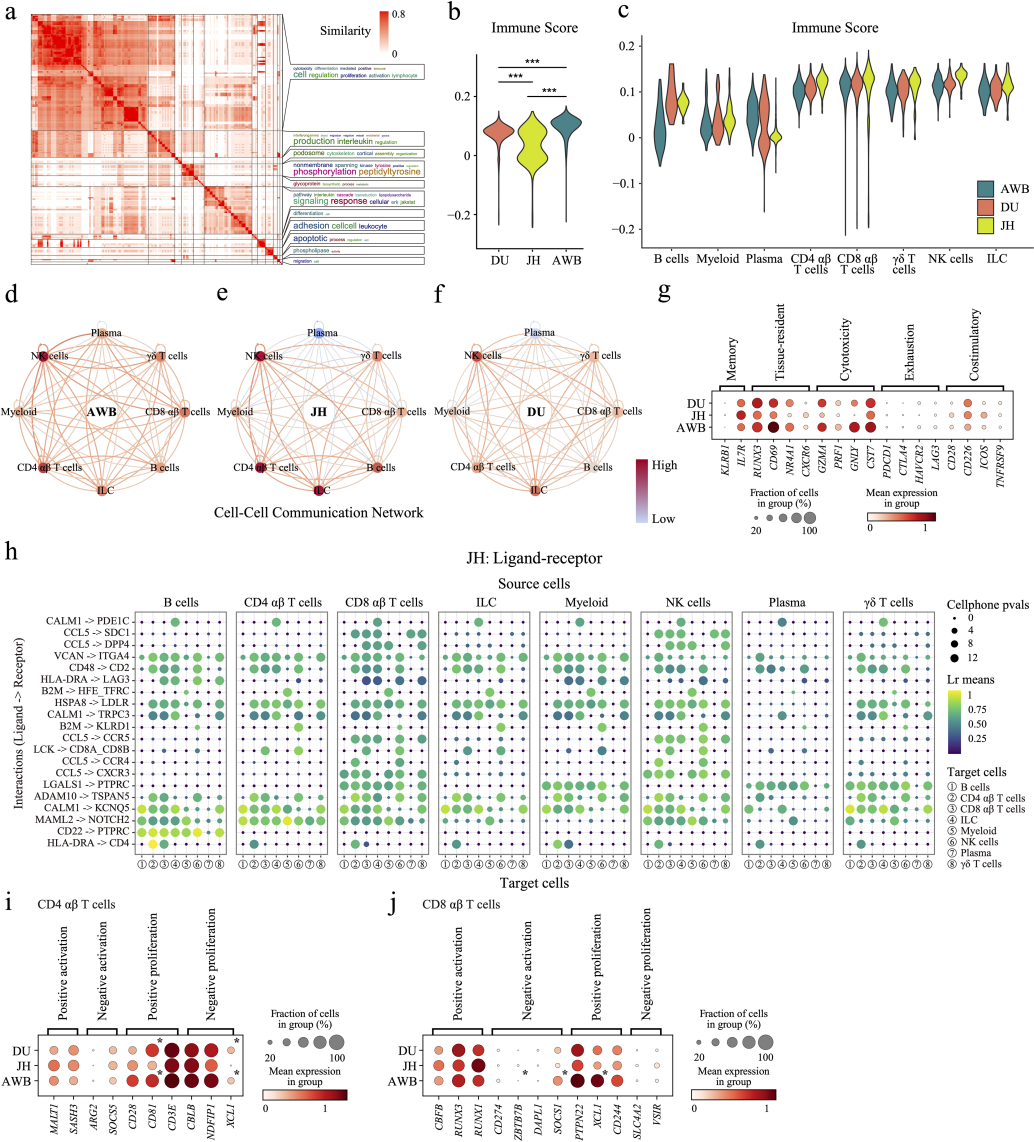
Differences in immune cells among the three pig species. **(a)** Clustering and word cloud analysis of GO enrichment results. **(b)** Scoring of immune-related genes in the immune cells of the three pig species. **(c)** Scoring of immune-related genes in different cell types. **(d–f)** Node colors indicate the relative strength of communication for each cell type, with darker colors representing higher average strength. Directed edges show the absolute strength of communication between cell types, with an edge from one type to another indicating their communication strength. Color intensity denotes relative communication intensity. **(g)** Dot plot displaying the expression levels of T-cell state-related genes in T/ILC/NK lymphocytes. **(h)** Dot plot of ligand-receptor interactions, where dot size represents the reversed cellphone p-value (larger dots indicate smaller p-values and stronger pathway specificity). Dot color indicates communication intensity, with colors closer to yellow-green indicating stronger communication. **(i, j)** Dot plot displaying the expression levels of T-cell proliferation and death related genes in CD4 αβ T cells **(i)** and CD8 αβ T cells **(j)**. Asterisk denotes significantly expression with FDR < 0.05 and |log2FC| > 1.5 compared with JH.

To compare immune levels among AWB, JH, and DU pigs, we identified 885 highly expressed genes in immune cells (**Supplementary Table S4**) and scored their immune levels (mentioned in the method). These genes were enriched in 191 immune-related Gene Ontology (GO) pathways, with most pathways linked to immunity, such as “immune”, “cytotoxicity”, and “lymphocyte” (**Figure 2a**, **Supplementary Table S5**). The immune scores (**Figure 2b**) and inflammatory scores (**Supplementary Figure S2b**) were significantly higher in AWB compared to JH and DU. But the immune scores in JH exhibited a bimodal distribution (**Figure 2c**). Further analysis within JH revealed that plasma cells also displayed bimodal distributions with notably high scores, suggesting greater subpopulation diversity (**Supplementary Figure S2k**). In contrast, AWB and DU showed more similar and uniform distributions (*p* = 0.14).

We also identified DEGs between immune cell subtypes (**Supplementary Table S6**) to infer cell type scores for different immune cell subpopulations. DU pigs generally exhibited the lowest scores, particularly in B cells, CD4 αβ T cells, myeloid cells, NK cells, and plasma cells. Conversely, JH and AWB had relatively higher scores across various immune cells, with AWB showing higher scores in B cells (**Supplementary Figure S2j**) and JH exhibiting elevated scores in plasma cells and myeloid cells (**Supplementary Figure S2g, k**). These findings suggest that JH has an enhanced capacity for immune regulation and defense via myeloid cells. Both AWB and JH demonstrate strong humoral immune functions, relying on B cells and plasma cells, respectively, while DU may exhibit comparatively weaker immune capabilities.

We assessed communication between immune cell subtypes by forecasting potential receptor-ligand interactions among the three pig breeds (Source Data). AWB demonstrated more balanced and stronger communication intensity among immune cells (**Figures 2d–f**). Additionally, JH plasma cells exhibited low communication intensity with other immune cells, while CD4 αβ T cells, CD8 αβ T cells, and NK cells showed high communication intensity (**Figure 2e**). Then, we ranked the average expression levels of ligand-receptor pairs and identified the top 20 key pairs with the highest expression (**Figure 2h**, **Supplementary Figure S2l, m**). These pairs showed conservation across breeds, including pathways such as B2M -> KLRD1, CD22 -> PTPRC, HSPA8 -> LDLR, CCL5 -> CCR4, CCL5 -> CCR5, CALM1 -> KCNQ5, ADAM10 -> TSPAN5, CD48 -> CD2, LCK -> CD8A_CD8B, LGALS1 -> PTPRC, CCL5 -> CXCR3, and MAML2 -> NOTCH2 were shared by all three pig breeds, the pathways CCL5 -> SDC1 and CALM1 -> PDE1C were shared by both JH and DU, the pathways TGFB1 -> TGFBR1_TGFBR2, CCL5 -> CCRL2, and TGFB1 -> CXCR4 were shared by both DU and AWB, and the pathways HLA-DRA -> CD4 were shared by both AWB and JH (**Figure 2h**, **Supplementary Figure 2l, m**). Most of these signaling pathways are crucial for immune responses and immune cell activation (63–67). They play crucial roles in NK cell recognition and activation (B2M -> KLRD1) (64), B-cell receptor signaling modulation (CD22 -> PTPRC) (65), T-cell and NK cell activation (CD48 -> CD2 and LCK -> CD8A_CD8B) (66, 67), and the recruitment of T-cells to inflammation sites (CCL5 -> CCR4, CCL5 -> CCR5, CCL5 -> CXCR3) (63). Other pathways are involved in cell differentiation and protein folding. The CALM1 -> KCNQ5 pathway is involved in calcium signaling, essential for cellular differentiation (68), while the ADAM10 -> TSPAN5 interaction plays a role in protein processing and cell adhesion, contributing to cellular differentiation and stability (69). However, we also found specificities in the pathway types and number of immune cells between the three pig breeds (**Figure 2h**, **Supplementary Figure 2l, m**). Notably, JH exhibited lower average expression of these key ligand-receptor pairs but engaged more pathways compared to AWB and DU (**Figure 2h**). This suggests that the complex cellular signaling in JH may compensate for its lower overall expression levels. AWB and DU showed greater similarity in their cellular communication pathways, indicating comparable molecular mechanisms of immune response. Additionally, plasma cells in JH exhibited specific signaling pathways, such as LGALS1 -> PTPRC and CD48 -> CD2, associated with T cell activation and apoptosis (66, 70). This indicates that the plasma cells in JH may possess unique characteristics and potentially more diverse functions compared to those in AWB and DU.

To further understand the cross-breed status of T cells, the largest immune cell population in this study (**Figure 1g**), we examined their functional characteristics. As key regulators of adaptive immunity, T cells play critical roles in coordinating immune responses, making them essential for understanding breed-specific immune strategies (71). Therefore, we analyzed the expression levels of markers associated with memory phenotype, tissue residency, exhaustion, cytotoxicity, and costimulatory functions (**Figure 2g**). Memory phenotype markers, such as *KLRB1* and *IL7R*, showed no significant differences in expression among three breeds, although JH had slightly higher expression levels (**Figure 2g**). Tissue-resident markers include *RUNX3*, *CD69*, *NR4A1*, and *CXCR6* (72–75) were highest in AWB, slightly higher in DU, and significantly lower in JH (**Figure 2g**). Cytotoxicity phenotype markers, including *GZMA*, *PRF1*, *GNLY*, and *CST7*, which are involved in the killing of infected cells and target cells such as tumors by T cells (76), followed a similar pattern to tissue residency markers—highest in AWB, slightly higher in DU, and significantly lower in JH (**Figure 2g**). Exhaustion phenotype markers, including *PDCD1*, *CTLA4*, *HAVCR2*, and *LAG3* (77), associated with T-cell functional inhibition and fatigue, were expressed at relatively low levels across all three pig breeds (**Figure 2g**). Costimulatory markers, including *CD28*, *CD226*, *ICOS*, and *TNFRSF9* (78), associated with T-cell activation and immune response, were slightly higher in AWB and DU compared to JH (**Figure 2g**). These results suggested that AWB exhibits the strongest T-cell-mediated immune functionality, followed by DU, while JH shows the weakest. Next, to investigate whether T cell function was affected by the activity of subtypes (mainly CD4 αβ cells and CD8 αβ cells), we identified the expression of genes related to proliferation of CD4 αβ T cells and CD8 αβ T cells, based on the GO database (79). In CD4 αβ T cells, the results showed that the expression levels of positive-proliferation-related genes (e.g. *CD81* and *CD3E*) and negative-proliferation-related genes (e.g. *NDFIP1* and *XCL1*) in JH were both significant lower compared to DU and AWB (**Figure 2i**). At the same time, the results showed that the expression levels of negative-activation-related genes (e.g. *ZBTB7B* and *SOCS1*) and positive-proliferation-related genes (e.g. *PTPN22* and *XCL1*) in JH were both significant lower compared to DU and AWB in CD8 αβ T cells (**Figure 2j**). These results implied a stable and balanced proliferation pattern of T cell in JH, even though the proportion of T cells in JH has a large variety heterogeneity.

### 3.4 Domestication-related breed-specific genes in immune cells

**Figure 3:**
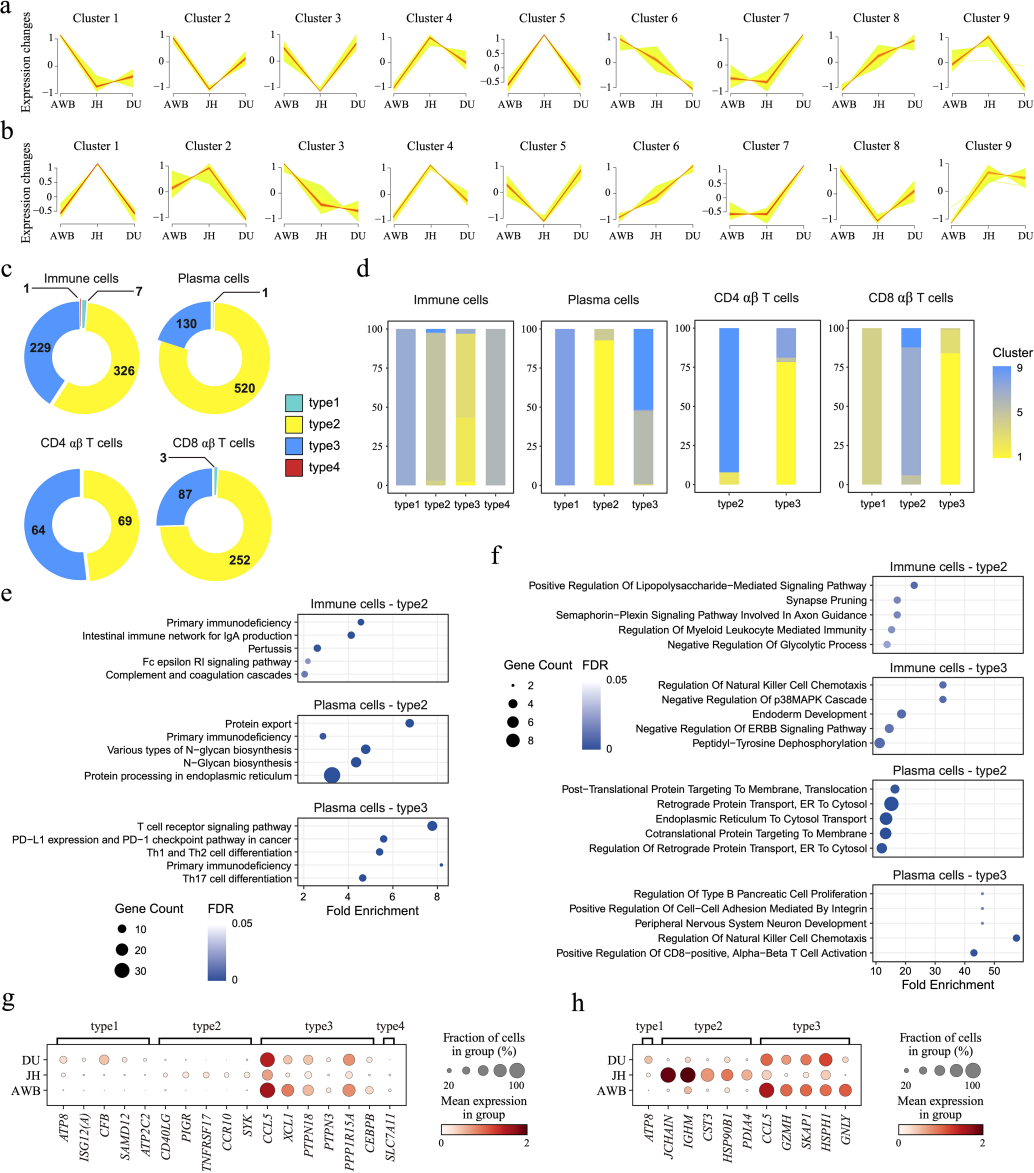
Changes in genes and gene patterns during domestication. **(a, b)** Fuzzy C-Means Clustering (FCM) analysis of genes, examining trends in gene expression over domestication time and clustering genes with similar expression patterns in in immune cells **(a)** and plasma cells **(b)**. **(c)** Pie chart of the proportion of gene sets with four different gene patterns in different cells. The number of genes is shown. **(d)** The intersection and source of four patterns genes in FCM results. **(e, f)** KEGG **(e)** and GO **(f)** enrichment analyses of four pattern genes in immune cells and plasma cells. **(g, h)** Dot plot showing the four gene patterns according to the domestication timeline in immune cells **(g)** and plasma cells **(h)**.

From wild boars to domestic local pigs and subsequently to intensive commercial pigs, the human-intervened domestication level continues to decrease (2, 4). To investigate the expression pattern of domestication-related genes on overall immune cells, we defined a domestication progression as moving from AWB to JH, and then to DU, then clustered genes with similar expression patterns along this fixed domestication progression (**Supplementary Table S7**) and performed enrichment analysis (**Supplementary Table S8**). In immune cells, the rising pattern genes (Cluster 8, 1,218 genes) throughout the domestication process were enriched in pathways related to metabolic diseases, such as maturity-onset diabetes of the young (**Figure 3a**). This may be related to the excessive energy intake in commercial pigs, leading to fat accumulation and subsequently triggering obesity-related metabolic issues (80). Conversely, genes with down pattern (Cluster 6, 1,395 genes) showed enrichment in pathways associated with cell division and proliferation, including nuclear membrane disassembly (**Supplementary Figure S3c**). This decline in expression may contribute to reduced immune capability in pigs under enhanced domestication levels.

We further explored the expression pattern of domestication-related genes in three cell types of immune cells in pigs: plasma cells, CD4αβ T cells, and CD8αβ T cells (**Supplementary Table S7**). In plasma cells, Cluster 3 (1,599 genes) and Cluster 6 (1,270 genes) represented gene sets with rising pattern and down pattern, respectively, along the domestication progression; however, these genes did not show significant enrichment (**Figure 3b**). In CD4αβ T cells, we identified rising pattern genes rather than down pattern genes along this fixed domestication progression (**Supplementary Figure 3a**), which were involved in protein synthesis and translational regulation (**Supplementary Figure 3d**), including processes of SRP-dependent co-translational protein targeting to membranes and processes of protein targeting to the endoplasmic reticulum (ER). These functions are crucial for maintaining cellular homeostasis by facilitating efficient protein processing and transport, particularly under stress conditions (81, 82). Additionally, these genes were associated with metabolic regulation pathways, such as insulin secretion and thyroid hormone synthesis (**Supplementary Figure 3d**), underscoring the role of intestinal immune cells in supporting the digestive and absorptive functions of organisms (83). In CD8αβ T cells, we observed down pattern genes along the domestication progression, whereas no gene clusters with rising patterns were identified (**Supplementary Figure 3b**). The down pattern genes were related to macromolecule metabolic and catabolic processes, such as proteasomal ubiquitin-independent protein catabolic processes (**Supplementary Figure 3e**). Reduced protein degradation can impair processes like cell cycle regulation and protein synthesis, limit the immune respond effectively ability of cells, and potentially disrupt cellular homeostasis and function (84). Moreover, these genes were also involved in energy metabolism pathways, including oxidative phosphorylation (**Supplementary Figure 3e**). Reduced energy production may damage the activation and maintenance ability of cells, potentially resulting in diminished immune response efficiency and weakened roles in immune surveillance and defense (85), especially in CD8αβ T cells from AWB to JH, and then to DU.

Furthermore, we classified DEGs into four distinct patterns based on the soft clustering classification of genes: the increasing expression pattern (type1), the JH-high expression pattern (type2), the JH-low expression pattern (type3), and the decreasing expression pattern (type4) (**Figures 3c, d**). These genes expressed obvious patterns under enhanced domestication levels and showed significant variety differences. In all immune cells, there was a large set of genes that were significantly JH-high-expressed or JH-low-expressed, which pointed to immune activities including primary immunodeficiency and signaling pathways including ERBB signaling pathway (**Figures 3e, g**). For plasma cells, the type1 pattern only had one gene, with the *ATP8* gene exhibiting a relatively clearer increasing trend (**Figure 3h**). *ATP8* gene encodes a subunit of the ATPase enzyme involved in ATP synthesis, which is critical for energy metabolism (86). A reduction in *ATP8* expression could indicate a decline in plasma cells’ energy-producing capacity, potentially impairing their ability to meet the high metabolic demands of antibody production. The type2 genes (520 genes) showed significant differences, such as *JCHAIN* and *IGHM* (**Figure 3f**), and were primarily involved in antibody production (87, 88). Type3 genes (130 genes) also exhibited significant differences, such as *CCL5*, *GZMH*, *SKAP1*, and *HSPH* genes (**Figure 3f**) play important roles in immune cell recruitment, activation, apoptosis, and stress response (89–92), implying JH plasma cells may have reduced functionality in cell communication and activation. Additionally, we also screened DEGs in CD4 αβ T cells and CD8 αβ T cells, such as *HBB*, *CCL5*, *CFB*, and *GNLY* (**Supplementary Figure 3f, g**). These significant genes helped us understand the domestication-related breed-specific immunology in immune cells, even though their association with domestication has not yet been confirmed. Despite the lack of significant expression level differences in type1 and type4 genes among the three pig breeds, these findings remain meaningful and can inform future studies with larger sample sizes (93).

### 3.5 Jinhua pig has a specific subtype of plasma cell populations

**Figure 4:**
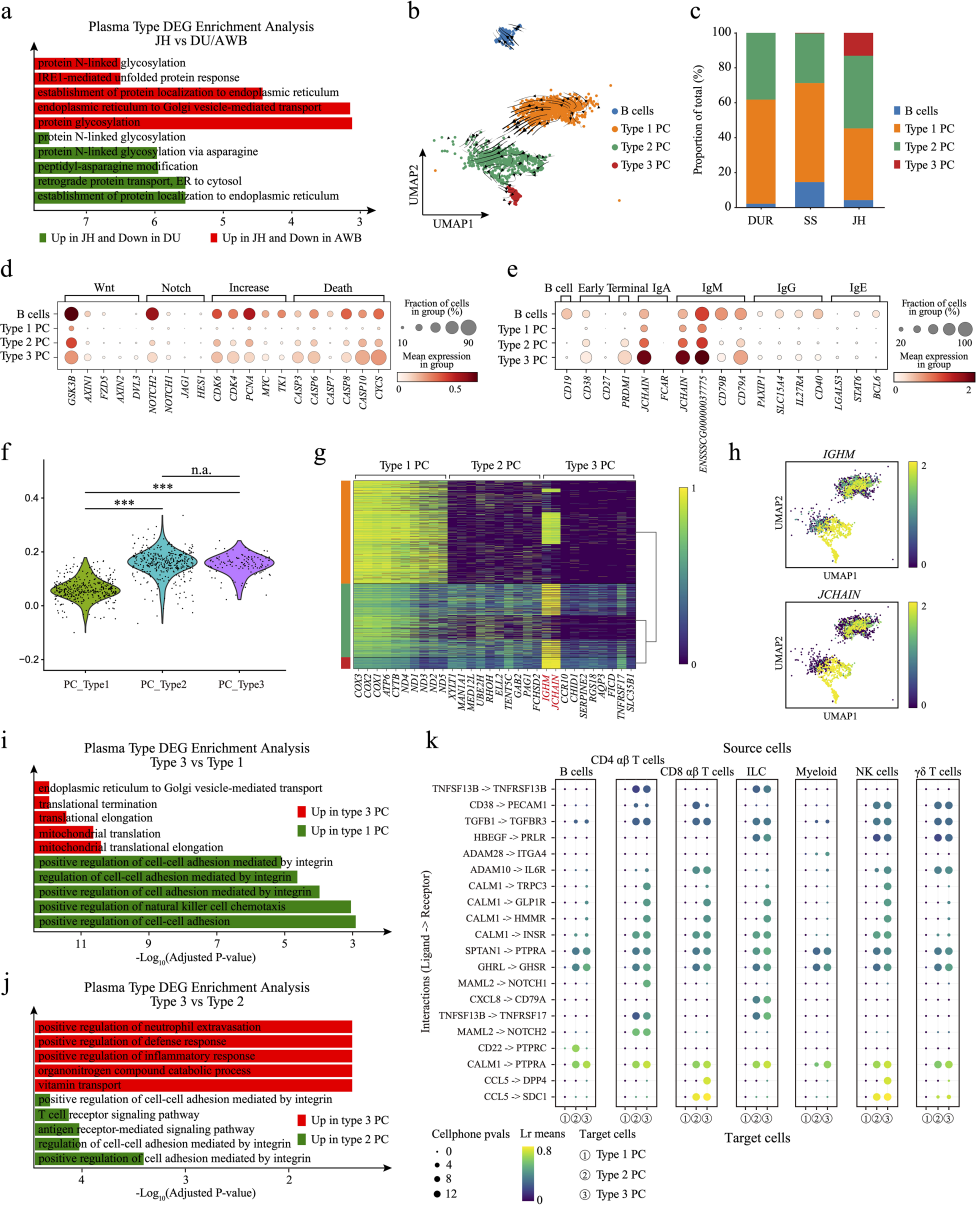
Exploration of plasma cell subpopulations. **(a)** Bar chart of DEG enrichment analysis. Red bars indicate enriched pathways for upregulated genes in JH compared to AWB, while green bars indicate enriched pathways for upregulated genes in JH compared to DU. **(b)** Velocity plot of plasma cells, with arrows indicating the direction of cell differentiation. **(c)** Bar plot showing the proportions of B cell and plasma cell subpopulations. **(d)** Dot plot displaying the differential expression of genes related to cell proliferation and apoptosis among different types of plasma cells. **(e)** Dot plot showing the expression levels of conventional plasma cell subtype marker genes across the three plasma cell types and B cells. **(f)** Plasma cell scores for the three types of plasma cells. **(g)** Heatmap of differentially expressed genes among the three types of plasma cells in the three pig species. **(h)** UMAP plots illustrating the expression of IGHM and JCHAIN genes in plasma cells. **(i)** Bar chart of DEG enrichment analysis. Red bars indicate enriched pathways for upregulated genes in type 3 plasma cells, while green bars represent enriched pathways for upregulated genes in type 1 plasma cells. **(j)** Bar chart of DEG enrichment analysis. Red bars indicate enriched pathways for upregulated genes in type 3 plasma cells, while green bars represent enriched pathways for upregulated genes in type 2 plasma cells. **(k)** Dot plot of ligand-receptor interactions, with the three plasma cell types serving as receptors. ***, p value < 0.001; n.a., p value > 0.05 (unpaired two-tailed Student’s t-test).

To investigate the heterogeneity in the proportion and regulatory mechanisms of plasma cells in JH, we performed pairwise comparisons of gene expression in plasma cells across three pig breeds. We identified DEGs for enrichment analysis (**Supplementary Table S9**). We found that the up-regulated genes in JH plasma cells were primarily associated with the proper folding, modification, and quality control of proteins within the endoplasmic reticulum. Key processes included protein N-linked glycosylation, protein N-linked glycosylation via asparagine, peptidyl-asparagine modification, and IRE1-mediated unfolded protein response (**Figure 4a**). These functions are crucial for the production of antibodies, as efficient processing and proper folding of immunoglobulins within the ER are essential (94–97). This indicates the presence of a plasma cell population in JH with a stronger capacity for antibody production compared to DU and AWB. Conversely, the down-regulated genes in JH plasma cells were enriched in intercellular communication (e.g. antigen receptor-mediated signaling pathway and T cell receptor signaling pathway) and cellular activity regulation (e.g. positive regulation of natural killer cell chemotaxis or positive regulation of cytokine production, **Supplementary Figure 4a**). This suggests that plasma cells in JH may have a diminished ability to regulate other immune cells compared to those in DU and AWB. When comparing the DEGs of plasma cells between AWB and DU, the enrichment analysis revealed that up-regulated genes in AWB were involved in the cellular response to interferon-gamma, such as cellular response to interferon-gamma pathway and interferon-gamma-mediated signaling pathway. In contrast, the up-regulated genes in DU plasma cells were associated with cell proliferation (e.g. regulation of developmental growth and positive regulation of developmental growth, **Supplementary Figure 4b**). These findings suggest that plasma cells from AWB and DU have more similar functional properties to each other than to those from JH, with active T cell activity in the AWB jejunum potentially being supported by plasma cells.

To explore the potential subtypes of plasma cells in JH, we performed Leiden clustering and identified three distinct clusters across the three pig breeds (**Figure 4b**). RNA velocity (**Figure 4b**) and pseudotime trajectory analysis (**Supplementary Figure 4c**) also suggested that the plasma cells landscape had a tripartite differentiation structure, with Type 1, Type 2, and Type 3 differentiation. Notably, we discovered a novel subtype, Type 3, characterized by a unique and highly differentiated plasma cell population exclusive to JH. This subtype demonstrates distinct gene expression profiles and functional attributes that differentiate it from plasma cell populations in AWB and DU. The Type 3 plasma cells constituted a significant proportion in JH (approximately 13.1%, **Figure 4c**). We re-examined the highly expressed genes in plasma cells and scored them. We found significant differences between Type 1 PC and both Type 2 and Type 3 in JH, which may explain the bimodal distribution of plasma cell scores observed in Jinhua pigs (**Figure 4f**). We examined the expression of genes involved in the Wnt (98) and Notch (99) signaling pathways, such as *GSK3B* and *NOTCH2*, within B cells and Type 3 plasma cells. These genes were highly expressed compared to other cell types (**Figure 4d**), indicating stronger cell growth and differentiation capabilities in B cells and Type 3 plasma cells. In contrast, Type 1 plasma cells exhibited the lowest expression levels of genes related to cell proliferation, including *CDK6*, *CDK4*, *PCNA*, *MYC*, and *TK1* (**Figure 4d**) (100–103). Additionally, genes involved in cell death regulation (104)—*CASP3*, *CASP6*, *CASP8*, *CASP10*, and *CYCS*—were also highly expressed in Type 3 plasma cells (**Figure 4d**), suggesting a higher turnover rate in this subtype. However, the pathway intensity in Type 2 plasma cells exhibited intermediate between Type 1 and Type 3 plasma cells.

To understand what types of plasma cells these three represent, we examined the marker genes of common plasma cell subpopulations (**Figure 4e**), The marker genes associated with common plasma cell subpopulations include those for B cells, such as *CD19* (105); early plasma cells, represented by *CD38* and *CD27* (106); and terminal plasma cells characterized by *PRDM1* (107). Additionally, specific antibody types are represented by IgA (*JCHAIN* and *FCAR*) (87, 108), IgM (*JCHAIN*, *IGHM*, *CD79B*, and *CD79A*) (87, 88, 109), IgG (*PAXIP1*, *SLC15A4*, *IL27RA* and *CD40*) (110–113), and IgE (*LGALS3*, *STAT6*, and *BCL6*) (114–116). It can be observed that Type 1 and Type 2 plasma cells did not show high expression of any specific state patterns; they primarily expressed plasma cell marker genes. In contrast, Type 3 plasma cells exhibited higher expression of genes associated with “long-lived,” “terminal,” and antibody secretion compared to Type 1 and Type 2. Based on previous classifications of human plasma cells, we speculate that Type 3 PCs are antibody-secreting plasma cells. However, it is challenging to determine the types of Type 1 and Type 2 plasma cells, possibly due to differences between human and pig plasma cell subclasses. Due to the lack of antibodies for stage-specific markers such as *CD38*, *CD138*, and *TACI* for humans and mice, plasma cells in the field of porcine immunology remain largely uncharacterized (117).

To understand the heterogeneity in gene expression and cellular function between Type 3 plasma cells and the other two subtypes in JH, we initially identified DEGs between three plasma cell types and performed functional enrichment analysis (**Figure 4g**, **Supplementary Table S10**). Type 1 plasma cells exhibited higher expression levels of mitochondrial genes, such as COX-family and ND-family genes (**Figure 4g**), indicating enhanced mitochondrial function (118, 119). The differential gene expression profiles of Type 2 and Type 3 plasma cells were more similar (**Figure 4g**), potentially reflecting an evolutionary relationship between these subtypes. Compared to Type 1 and Type 2 plasma cells, Type 3 plasma cells maintained more stable and high expression of *JCHAIN* and *IGHM* genes (**Figures 4g, h**). The DEG analysis between Type 2 and Type 3 in JH further identified specific genes, such as *PRDX4* and *ATP5PO*, uniquely expressed in Type 3 plasma cells (**Supplementary Figure 4d**). Functional enrichment revealed that the up-regulated genes in Type 3 plasma cells were enriched in pathways related to protein transport (e.g. endoplasmic reticulum to Golgi vesicle-mediated transport), translation mechanisms (e.g. translational termination and translational elongation), and mitochondrial functions (e.g. mitochondrial translational elongation, **Figure 4i**). These pathways are critical for antibody production in plasma cells. In contrast, Type 1 plasma cells showed enrichment in cellular regulatory functions such as positive regulation of cell-cell adhesion and positive regulation of natural killer cell chemotaxis (**Figure 4i**). When comparing Type 2 and Type 3 plasma cells, the up-regulated genes in Type 3 plasma cells were associated with immunity (e.g. positive regulation of defense response and positive regulation of inflammatory response), whereas Type 2 up-regulated genes were involved in cell adhesion and communication (e.g. antigen receptor-mediated signaling pathway and positive regulation of cell-cell adhesion mediated by integrin, **Figure 4j**).

Analysis of cell communication revealed that the communication strength between plasma cells and other immune cells was generally low, with Type 3 plasma cells exhibiting the weakest interactions (**Supplementary Figure 4f** and Source Data). We speculated that this reduced communication strength may be due to a decrease in cell-cell adhesion proteins, such as cell adhesion molecules or connexins, in Type 3 plasma cells (**Figure 4i**). This reduction could potentially affect or disrupt the cell adhesion and channel connections between cells (120). To validate this hypothesis and elucidate the potential differentiation mechanisms of Type 3 plasma cells, we investigated the top 20 highly expressed ligand-receptor pairs (**Figure 4k**, **Supplementary Figure 4g**). When acting as signaling senders, Type 2 and Type 3 plasma cells shared similar ligand-receptor interactions but exhibited greater heterogeneity compared to Type 1 plasma cells (**Supplementary Figure 4g**). Notably, some cell migration (121–123) signaling pathways such as *LGALS1*->*PTPRC* and *CD40LG*->*CD53* in Type 3 plasma cells and *TFF3*->*CXCR4* in Type 2 plasma cells, exhibited cell specificity (**Supplementary Figure S4g**). When acting as the signaling receivers, Type 3 plasma cells engaged extensively in a cell communication pathway mediated by calcium-binding protein *CALM1*, interacting with targets such as *TRPC3*, *GLP1R* or *HMMR* (**Figure 4k**). Additionally, Type 3 plasma cells received the specific CCL5 -> DPP4 signal from CD8 αβ T cells and the MAML2 -> NOTCH1 signal from both CD4 and CD8 T cells. These pathways are crucial for regulating the differentiation of progenitor cells into specialized cell types, influencing cell development and differentiation (124, 125). To further confirm the authenticity of the cell communication signals, we conducted a gene regulatory network (GRN) analysis (Source Data). We found that transcription factors such as *BHLHA15*, *CUX1*, *MEF2C*, *IRF4*, and *PRDM1* were highly expressed in Type 3 plasma cells (**Supplementary Figure S4h** and Source Data), as indicated by literature regarding their important roles in cell differentiation (126–130). By integrating the cell communication results with the GRN analysis, we discovered that the *NOTCH1* gene is significantly regulated by *PRDM1* (**Supplementary Figure S4i**). We speculate that these may serve as potential signals promoting the differentiation of Type 3 plasma cells. Overall, Type 3 plasma cells represent a functional subtype unique to JH, characterized by enhanced protein processing and antibody secretion capabilities.

## 4 Discussion

Previous studies have primarily employed low-resolution approaches to analyze different pig breeds, including evolutionary and gene regulatory analyses (2–4). In our study, we addressed these issues by utilizing single-cell transcriptomics to construct a detailed atlas of immune cells in the jejunum of pigs at various domestication stages. This approach allowed us to uncover distinct immune cell populations and their specific gene expression profiles, enhancing our understanding of breed-specific immune adaptations. By revealing these nuanced differences, our findings contribute to the development of targeted breeding strategies aimed at improving disease resistance and overall health in domestic pigs. In the field of porcine immunology, antibody-secreting plasma cells have largely remained uncharacterized (117). Our study also focused on plasma cells, delving deeply into their characteristics, and identifying interbreed differences. This provides insights for future research on pig plasma cells.

In this study, we constructed a single-cell atlas of jejunal tissues from pigs at various domestication stages: wild boars, a Chinese local breed (JH), and an intensive breed (DU). We comprehensively investigated the functional heterogeneity of their cells. Using single-cell transcriptomics, we mapped the immune and epithelial cells in the jejunum of pigs, revealing significant differences in cell type composition and immune cell ratios between AWB, JH, and DU. Notably, our analysis showed a higher proportion of immune cells and a lower proportion of fibroblasts and endothelial cells compared to previously described small intestine compositions (131). For example, the proportion of T/ILC/NK cells was relatively high as previously reported (132). This discrepancy may reflect biological heterogeneity or result from challenges in cell dissociation and incomplete capture (133). We agreed that differences in cell numbers and sequencing depth can affect the expression patterns in scRNA-seq matrix (134–136). We computed the correlation between downsampled matrix and our current matrix, and the final value reached 0.9 and above, porting the strategy of continuing to use the original data for analysis. The information loss caused by downsampling may affect the representativeness and stability of our results (137). Despite limitations of our high-quality single-cell RNA sequencing, such as batch effect in cell numbers and sequencing depth, our study provides a robust cellular gene expression profile without downsampling. Marker gene analysis revealed that AWB and JH pigs exhibited higher expression of B cell markers, whereas DU pigs showed elevated expression of epithelial cell markers. The enhanced immune marker expression in AWB and JH pigs is likely a result of their exposure to diverse pathogens in natural environments (138, 139), driving the evolution of robust immune systems and increased disease resistance (140). In contrast, commercial Duroc pigs, bred in stable environments under artificial selection (141), demonstrate resupduced immune adaptations but improved growth and nutrient absorption capabilities (142).

Previous studies have used DEGs for enrichment and scoring enriched gene sets to assess differences in cellular functional states (143, 144). We referenced and improved this method by scoring immune cell functional levels based on DEG sets. B cells in AWB and JH pigs showed higher immune scores and stronger humoral immunity, whereas DU had lower B cell scores and potentially weaker immune capacity. Consistent with our conclusion, previous studies have shown that commercial pigs like DU have undergone intense artificial selection for production traits, while some indigenous Chinese pig breeds have mainly been shaped by natural selection throughout their long domestication to enhance immunity and disease resistance rather than to increase productivity (142). Additionally, immune cell communication pathways, such as B2M->KLRD1 and CD22->PTPRC (64, 65), were conserved across breeds, while JH pigs exhibited unique pathways like LGALS1->PTPRC and CD48->CD2 in plasma cells (66, 70). We hypothesize that JH’s distinct immune mechanisms may result from multiple domestication centers in China (145) and its long history of independent breeding (146), leading to early differentiation from AWB and contributing to notable molecular differences compared to both DU and AWB. We also found that plasma cells in JH pigs differ significantly from those in AWB and DU, further highlighting their unique gene expression profile. We reported that AWB and DU displayed the higher marker genes expression of tissue residency and cytotoxic than JH, and stable function of cell proliferation in our adult JH. This result may point to the heterogeneous proportion of CD4/CD8 αβ T cells in our adult JH. As reported, genetics, environment, and developmental stages may contribute to differences in T cell populations. The quantity and function of T cells can change (147) with developmental stages until reaching equilibrium in adulthood (148). Moreover, genetic differences between breeds could influence immune tolerance (149), immune-regulatory genes (150) and the timing of immune homeostasis (151), resulting in differing T cells across breeds. We speculated that the proportion changes of T cells in JH may occur at a younger developmental stage, to respond to infections or other immune challenges. This timeline changes of the immune in JH needs more experimental verification in the future.

The enhanced domestication levels from AWB to JH and then to DU pigs led to breed-specific genes characterization, which related to metabolic diseases or cell division in immune cells. This pattern implies that domestication prioritized rapid growth over immune function (152), a trade-off also influenced by farming practices that reduce immune system stimulation to prevent appetite suppression (153). Specifically, energy metabolism-related genes in CD8 αβ T cells and *APT8* gene in overall immune cells highlighting the importance of cytotoxic activity (154) and energy metabolism (86), under varying domestication levels. Such a domestication process of the immune function will also enable comparison with human self-domestication (155) although further method development may be required to map cross-species datasets.

Importantly, we identified a unique subtype of plasma cells in Jinhua pigs characterized by high and stable expression of *JCHAIN*, *IGHM*, *PRDX4*, and *ATP5PO* genes, alongside active protein synthesis and cell proliferation regulation. These plasma cells exhibited reduced adhesive capabilities and altered cell communication signals, receiving distinct signals such as CCL5->DPP4 from CD8 αβ T cells and MAML2->NOTCH1 from both CD4 and CD8 T cells, which are crucial for B cell activation (125) and progenitor cell differentiation (124). We also found that the expression of *NOTCH1* is associated with the type 3 plasma cells’ specific transcription factor *PRDM1* (130), which is closely related to plasma cell differentiation. This unique plasma cell subtype underscores breed-specific immune adaptations, offering insights into enhancing immune responses through selective breeding strategies.

The limitations of our study are the small sample size and vary experimental design, which may cause dataset-specific batch effects and affect the precision of proportion assessments and the representativeness of the findings. Previous high-quality research has acknowledged that multiple-sample RNA-Seq datasets can provide more information and reduce errors compared to single-sample datasets (156). We validated key findings such as cell dominance using immunofluorescence in the jejunum in separate cohorts of pigs. However, this validation was not applicable to all research conclusions in our study. In the future, collecting a larger sample size and different breeds of pig jejunum samples will enable us to determine the extent of heterogeneity in intestinal cells. As this project focused on immune function, the obtained immune cells are sufficient for identifying potential cell-cell interaction and target genes. We find a novel plasma cell group in the jejunum of JH. However, due to differences in cell numbers among the three breeds, we are currently unable to verify whether this cell group is completely absent in DU and AWB. Future experiments of capturing and separating plasma cell subclusters will be of considerable interest. In summary, our results provide new insights into characterizing the intestinal immune system of pigs.

In conclusion, we constructed a single-cell atlas of jejunal tissues from pigs with different domestication statuses and comprehensively investigated the functional heterogeneity of their cells. Our results indicated that the diversity immunological differentiation during the domestication process from Asian wild boar to a Chinese local pig and then to an intensive pig. Further, we identified a unique subtype of plasma cells in Jinhua pigs. Overall, these findings deepen our understanding of pig immunology and inform breeding strategies aimed at improving disease resistance and overall health in livestock.

## Code availability

The code generated during this study is available at Github: https://github.com/Wenyu-Fu/Pig-Jejunal-Single-Cell-RNA-Landscapes.

## Data Availability

The datasets presented in this study can be found in online repositories. The names of the repository/repositories and accession number(s) can be found in the article/**Supplementary Material**.
