# Corrigendum: Pig jejunal single-cell RNA landscapes revealing breed-specific immunology differentiation at various domestication stages

**DOI:** 10.3389/fimmu.2025.1588642

**Published:** 2025-03-13

**Authors:** Wenyu Fu, Qinqin Xie, Pengfei Yu, Shuang Liu, Lingyao Xu, Xiaowei Ye, Wei Zhao, Qishan Wang, Yuchun Pan, Zhe Zhang, Zhen Wang

**Affiliations:** ^1^ College of Animal Sciences, Zhejiang University, Hangzhou, China; ^2^ SciGene Biotechnology Co., Ltd, Hefei, China; ^3^ Hainan Institute of Zhejiang University, Building 11, Yongyou Industrial Park, Yazhou Bay Science and Technology City, Yazhou District, Sanya, China; ^4^ Key Laboratory of Livestock and Poultry Resources Evaluation and Utilization, Ministry of Agriculture and Rural Affairs, Hangzhou, China; ^5^ Hainan Yazhou Bay Seed Lab, Yongyou Industrial Park, Yazhou Bay Sci-Tech City, Sanya, China

**Keywords:** single-cell RNA sequencing, jejunum, immune cells, domestication, plasma cells

In the published article, there was an error in the **Funding** statement. The incorrect grant number (32378231) was given for the National Natural Science Foundation of China. The correct **Funding** statement appears below.

“The author(s) declare that financial support was received for the research, authorship, and/or publication of this article. This work was financially supported by Zhejiang Provincial Natural Science Foundation of China (Grant number: LZ23C170003), the National Natural Science Foundation of China (Grant number: 32372831), the National Key Research and Development Program of China (Grant number: 2021YFD1200802, 2023YFF1001100, and 2022YFF1000500), and Zhejiang Science and Technology Major Program on Agricultural New Variety Breeding (Grant number: 2021C02068-1 and 2021C02068-2).”

The authors apologize for this error and state that this does not change the scientific conclusions of the article in any way. The original article has been updated.

