# Supplementary material for: Pig jejunal single-cell RNA landscapes revealing breed-specific immunology differentiation at various domestication stages
Source: Front Immunol. 2025 Feb 28;16:1530214. doi: 10.3389/fimmu.2025.1530214 (PMC11947726; doi:10.3389/fimmu.2025.1530214)
Supplement: Supplementary file 1 [file DataSheet1.docx]

# Pig Jejunal Single-Cell RNA Landscapes Revealing Immunological Differentiation Shaped by Domestication

Wenyu Fu^1,†^, Qinqin Xie^1,†^, Pengfei Yu^1^, Shuang Liu^1^, Lingyao Xu^1^, Wei Zhao^5^, Qishan Wang^1,2,4^, Yuchun Pan^1,2,3,4^, Zhe Zhang^1,4,*^, Zhen Wang^1,4,*^

^1^College of Animal Sciences, Zhejiang University, Hangzhou 310058, P.R. China

^2^Hainan Institute of Zhejiang University, Building 11, Yongyou Industrial Park, Yazhou Bay Science and Technology City, Yazhou District, Sanya 572025, P.R. China

^3^Hainan Yazhou Bay Seed Lab, Yongyou Industrial Park, Yazhou Bay Sci-Tech City, Sanya 572025, P.R. China

^4^Key Laboratory of Livestock and Poultry Resources Evaluation and Utilization, Ministry of Agriculture and Rural Affairs, Hangzhou 310058, P.R. China

^5^SciGene Biotechnology Co., Ltd, Hefei 230031, P.R. China

*Corresponding authors

^†^Wenyu Fu and Qinqin Xie contributed equally to this work.

**Corresponding authors:**

Zhen Wang, E-mail: wangzhen20@zju.edu.cn; Zhe Zhang, E-mail: zhe_zhang@zju.edu.cn

**E-mails for all authors:**

Wenyu Fu: 786967870qq@gmail.com

Qinqin Xie: qinqin.xie@zju.edu.cn

Pengfei Yu: pengfei_yu@zju.edu.cn

Shuang Liu: 22117005@zju.edu.cn

Lingyao Xu: xulingyao@zju.edu.cn

Qishan Wang, E-mail: wangqishan@zju.edu.cn

Yuchun Pan, E-mail: panyc@zju.edu.cn

Zhe Zhang, E-mail: zhe_zhang@zju.edu.cn

Zhen Wang, E-mail: wangzhen20@zju.edu.cn

**
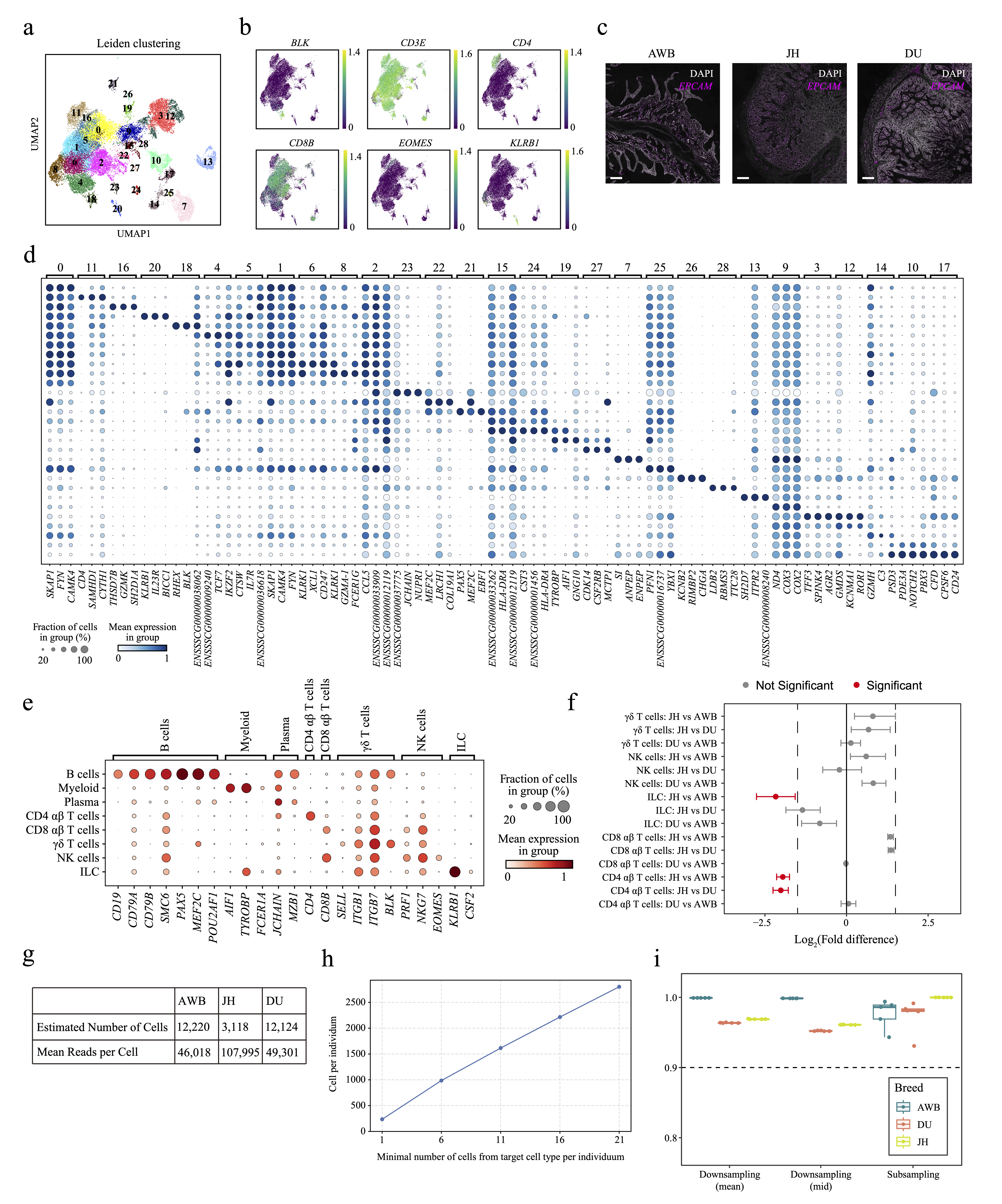
**

**Supplementary Fig. 1| Supplementary clustering methods.**

**a,** The initial Leiden clustering resulted in 28 clusters.

**b,** UMAP plot showing the expression of 6 marker genes for immune cell types, with yellow-green indicating high expression.

**c,** Immunofluorescence visualization of the jejunum using markers for *EPCAM*+ epithelial cells.

**d,** Differential genes of the 28 clusters from the initial Leiden clustering.

**e,** Dot plot displaying marker genes expressed in 8 types of immune cells.

**f,** Relative differences in cell proportions for each cluster between different breeds in all immune cells. Red clusters have an FDR < 0.05 and mean |log_2_(Fold difference)| > 1.5 under comparison (permutation test; n = 1,000).

**g,** Table of estimated number of cells and mean reads per cell of original data grouped by pig breeds.

**h,** Line chart showing the required number of cells per individual (y-axis, log scale) to detect the minimal number of cells from a target cell type per individuum (x-axis) with a certain probability. The minimal number of cells to range from 1 to 21.

**i,** Box plot of the correlation value between the downsampled data and the original data.


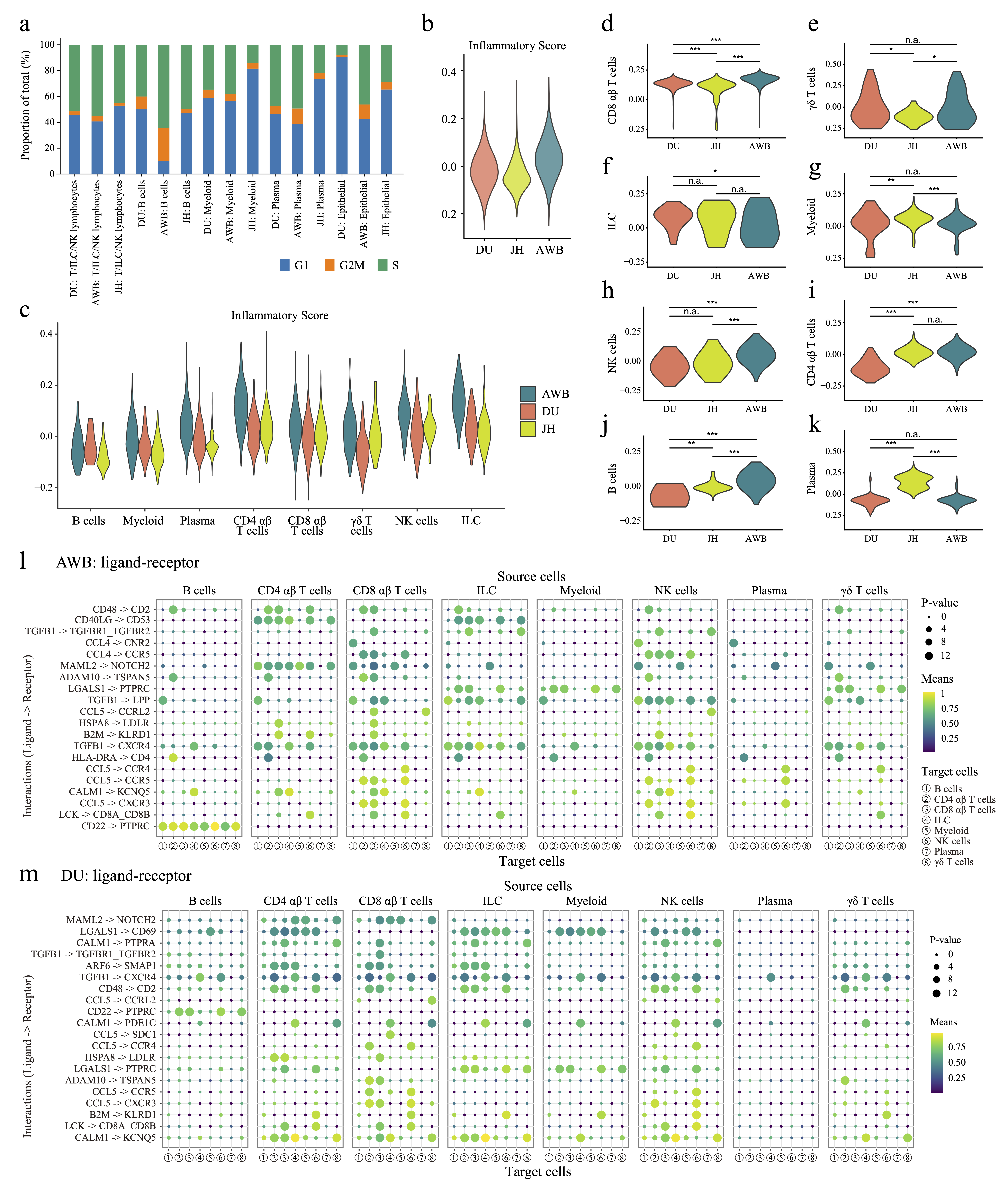


**Supplementary Fig. 2| Cell score and ligand-receptor analysis.**

**a,** Proportions of cells in various cycle stages.

**b,** Scoring of inflammatory-related genes in immune cells of the three pig species.

**c,** Scoring of inflammatory-related genes across different cell types.

**d-k**, Scoring of immune-related genes in the 8 types of immune cells among the three pig species.

**l-m,** Dot plot of ligand-receptor interactions for AWB (**l**) and DU (**m**), where dot size represents the reversed cellphone p-value (larger dots indicate smaller p-values and stronger pathway specificity).


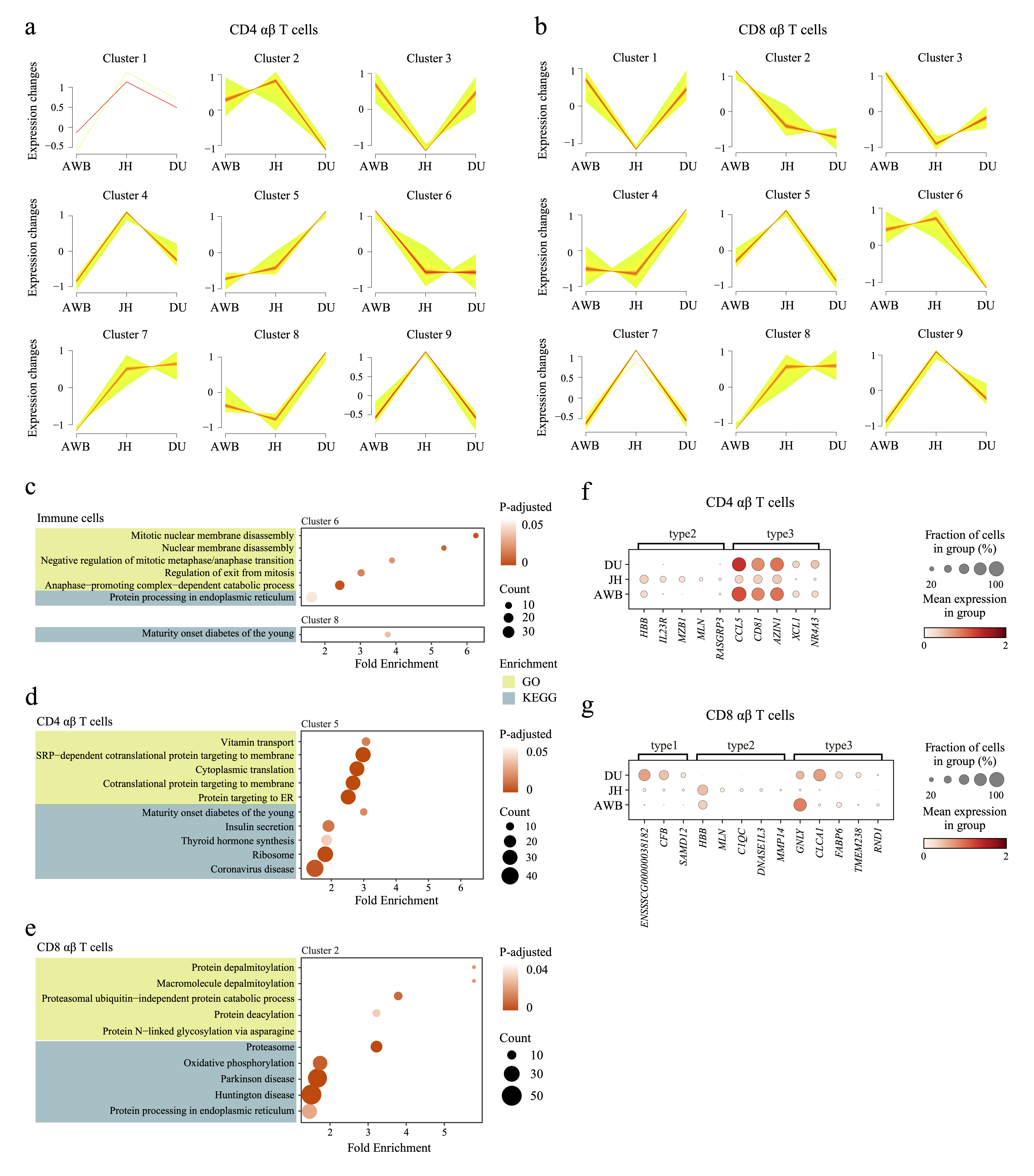

**Supplementary Fig. 3| Changes in genes and gene patterns during domestication.**

**a-b,** Fuzzy C-Means Clustering (FCM) analysis of genes, examining trends in gene expression over domestication time and clustering genes with similar expression patterns in CD4 αβ T cells (**g**) and CD8 αβ T cells (**h**).

**c-e,** GO and KEGG enrichment analyses of genes clustered based on increasing and decreasing trends during domestication in immune cells (**c**), CD4 αβ T cells (**d**) and CD8 αβ T cells (**e**).

**f-g,** Dot plot showing the four gene patterns according to the domestication timeline in CD4 αβ T cells (**f**) and CD8 αβ T cells (**g**).


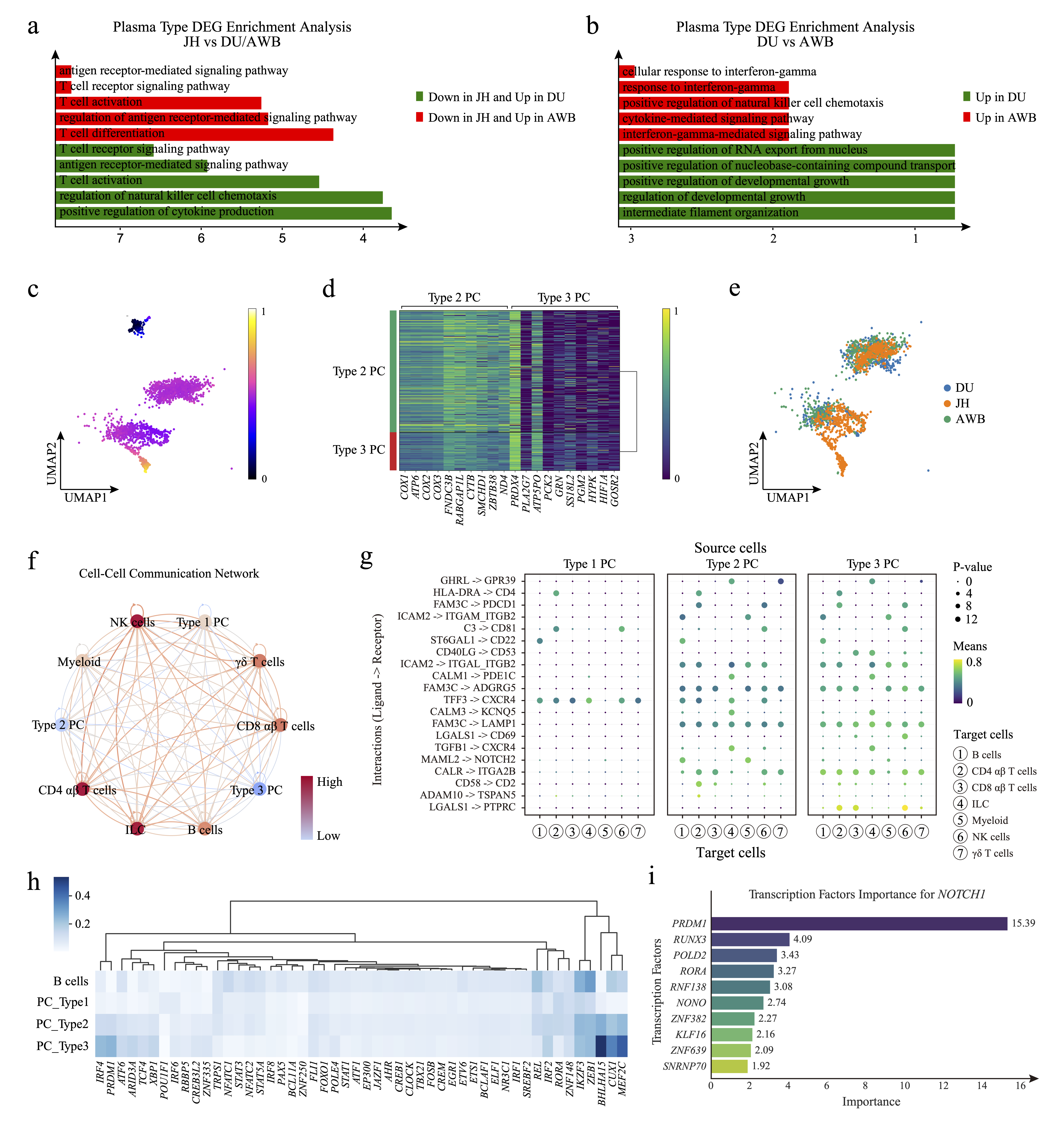


**Supplementary Fig. 4| Exploration of plasma cell subpopulations.**

**a,** Bar chart of DEG enrichment analysis. Red bars indicate enriched pathways for downregulated genes in JH compared to AWB, while green bars represent enriched pathways for downregulated genes in JH compared to DU.

**b,** Bar chart of DEG enrichment analysis. Red bars indicate enriched pathways for upregulated genes in AWB compared to DU, while green bars represent enriched pathways for upregulated genes in DU compared to AWB.

**c,** Pseudotime analysis (darker colors represent earlier pseudotime, lighter colors represent later pseudotime).

**d,** Heatmap of differential genes between Type 2 and Type 3 plasma cells in JH.

**e,** UMAP plot showing the specific distribution of Type 3 plasma cells in JH.

**f,** Cell-cell communication network revealing communication intensity between the three plasma cell types and seven immune cell types.

**g,** Dot plot of the top 20 ligand-receptor interactions when the three plasma cell types act as ligands.

**h,** Heatmap of GRN analysis for the three plasma cell types, with darker blue indicating higher estimated activity of transcription factors (TFs).

**i,** The top 10 TFs with the highest estimated activity for the *NOTCH1* gene, with higher values indicating greater estimated activity.
